# Biomarkers of postoperative delirium and cognitive dysfunction

**DOI:** 10.3389/fnagi.2015.00112

**Published:** 2015-06-09

**Authors:** Ganna Androsova, Roland Krause, Georg Winterer, Reinhard Schneider

**Affiliations:** ^1^Bioinformatics core, Luxembourg Centre for Systems Biomedicine (LCSB), University of LuxembourgBelvaux, Luxembourg; ^2^Experimental and Clinical Research Center (ECRC), Department of Anesthesiology and Operative Intensive Care Medicine, Charité University Medicine BerlinBerlin, Germany

**Keywords:** postoperative delirium, postoperative cognitive dysfunction, biomarker, systems biology, POD, POCD

## Abstract

Elderly surgical patients frequently experience postoperative delirium (POD) and the subsequent development of postoperative cognitive dysfunction (POCD). Clinical features include deterioration in cognition, disturbance in attention and reduced awareness of the environment and result in higher morbidity, mortality and greater utilization of social financial assistance. The aging Western societies can expect an increase in the incidence of POD and POCD. The underlying pathophysiological mechanisms have been studied on the molecular level albeit with unsatisfying small research efforts given their societal burden. Here, we review the known physiological and immunological changes and genetic risk factors, identify candidates for further studies and integrate the information into a draft network for exploration on a systems level. The pathogenesis of these postoperative cognitive impairments is multifactorial; application of integrated systems biology has the potential to reconstruct the underlying network of molecular mechanisms and help in the identification of prognostic and diagnostic biomarkers.

## Introduction

**Figure 1:**
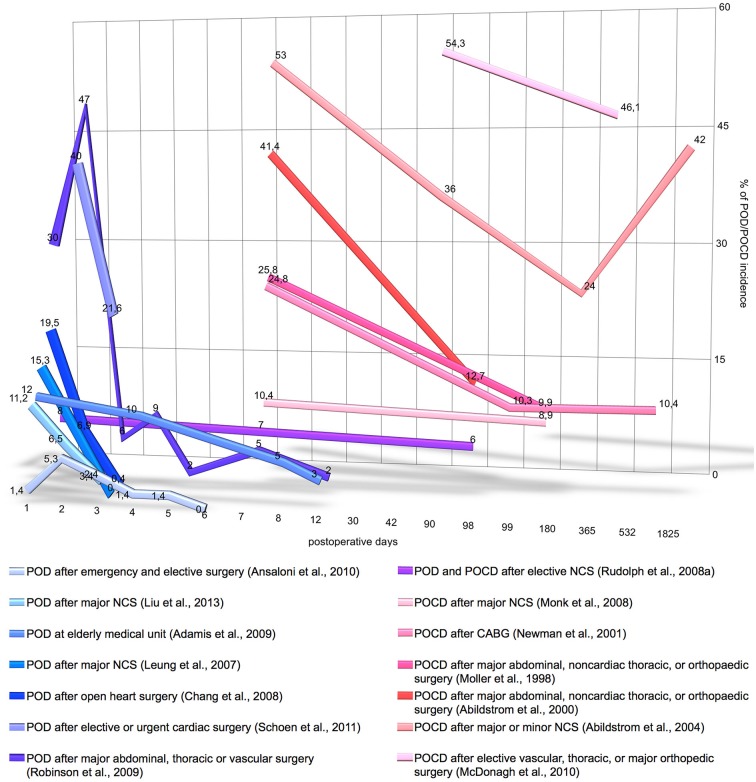
**Incidence and time-course of postoperative delirium (POD) and postoperative cognitive dysfunction (POCD) incidence**. *Y*-axis denotes the percentage on POD/POCD incidence registered by the different studies. *X*-axis denotes the number postoperative days on logarithmic scale. The graph does not include the data of POD/POCD incidence, if it was measured only once postoperatively, if measurement time was not precisely stated or the study includes less than 140 patients. CABG, coronary artery bypass grafting; NCS, noncardiac surgery.

More than 40% of surgical procedures in the US are performed on patients aged 65 and over Control Prevention Centers for Disease (2010). Elderly patients frequently experience postoperative cognitive impairment, characterized by progressive cognitive and sensory decline. An acute phase of cognitive impairment is postoperative delirium (POD; according to DSM-5: 293.0 “Delirium Due to Another Medical Condition”; Rudolph et al., 2008a). Deliria are further classified by duration and level of activity such as hyperactive, hypoactive or mixed. Patients with POD frequently develop a chronic phase of cognitive impairment, i.e., postoperative cognitive dysfunction (POCD; according to DSM-5: 294.10/11 “Major Neurocognitive Disorder Due to Another Medical Condition Without/With Behavioral Disturbance” or 331.83 “Mild Neurocognitive Disorder Due to Another Medical Condition”; Rudolph et al., 2008a). POCD is developed in 32% of patients with short delirium duration (1–2 days) and in 55% of patients with longer delirium (Rudolph et al., 2008a). The incidence of POD/POCD varies depending on the study and type of surgery; as illustrated on Figure 1, POD incidence ranges from 13.2% to 41.7% and POCD incidence ranges from 8.9% to 46.1%. The prevalence of POD and POCD is associated with higher mortality, increased incidence of postoperative complications, longer duration of hospital stay, greater utilization of social financial assistance and earlier retirement (Greene et al., 2009; Robinson et al., 2009; Steinmetz et al., 2009; Ansaloni et al., 2010; Liu et al., 2013). Patients older than 65 are predisposed to POD and POCD if they have hypoalbuminemia, abnormal preoperative serum sodium, potassium, glucose or blood sugar levels as well as psychopathological symptoms, alcohol abuse or co-morbidities (Moller et al., 1998; Abildstrom et al., 2000; Newman et al., 2001; Chang et al., 2008; Monk et al., 2008; Deiner and Silverstein, 2009; Ansaloni et al., 2010; Kazmierski et al., 2014b). The cognitive status of elderly patients including depression, dementia or cognitive impairment is a significant risk factor for development of POD (Elie et al., 1998; Leung et al., 2005; Minden et al., 2005; Inouye, 2006; McAvay et al., 2007; Greene et al., 2009; Kosar et al., 2014). For instance, dementia is a significant risk factor that increases delirium occurrence risk by fivefold (Elie et al., 1998);* vice versa*, delirium itself may lead to dementia and long-term cognitive deterioration (Jackson et al., 2004).

Postoperative delirium is defined in DSM-5 by several criteria including clouding of consciousness with reduced awareness of environment and difficulty in sustaining and/or shifting attention. In addition, POD is characterized by changes in cognition that affect memory, language and orientation in time/space (American Psychiatric Association, 2013). The impairment of memory, perceptual-motor abilities, language and attention are transit characteristics between POD and POCD. Memory impairment significantly affects cognitive decline and leads to impaired social and professional functioning in POCD patients. Memory deterioration lasting more than 1 month signifies the entry into the chronic phase of the cognitive impairment (American Psychiatric Association, 2013). POCD following delirium might increase the rate of cognitive deterioration in Alzheimer’s disease (Gross et al., 2012). Specific characteristics of POCD include decline in speed of processing the information and disturbance in executive functioning but the patient typically remains oriented to person, time and space (Tsai et al., 2010). The decline in POCD is mostly recognized by comparison to the patient’s pre-operative capabilities (Deiner and Silverstein, 2009). Delirium is usually measured by standardized clinical tests such as the Confusion Assessment Method (Inouye et al., 1990).

To improve diagnosis and treatment of POD/POCD, research aimed to identify prognostic and diagnostic biological markers. Biomarkers can determine severity and phase of the cognitive impairment, stratify patients who are likely to respond to specific treatment and monitor the efficiency of the treatment. Genetic markers (Papadopoulou et al., 2006), RNA (Sørensen and Ørntoft, 2010) and microRNA (Scherzer et al., 2007) levels, proteins (Wang et al., 2005), and post-translational changes such as glycosylation (Norton et al., 2008; Drake et al., 2010) and phosphorylation (Deguchi et al., 2002), have been demonstrated as prognostic biomarkers in a variety of diseases including disorders of the central nervous system (CNS) and these biochemical entities should be considered as possible markers for POD and POCD.

The most prominent hypothesis for the molecular mechanisms of POD and POCD is a central cholinergic deficiency caused by deregulation of cholinergic anti-inflammatory pathways leading to increased inflammation (Inouye, 2006). Despite detection of decreased acetylcholine levels, several studies reported contradictory findings regarding levels of serum anticholinergic activity (SAA; see Section Biological Markers of Postoperative Delirium). Another suggestion is that delirium is caused by a combination of dopamine excess and acetylcholine deficiency (Trzepacz, 2000). Low tryptophan levels can be associated with delirium via decreased synthesis of brain serotonin or alteration of melatonin production, which has been challenged (see Section Biological Markers of Postoperative Delirium). The association between POD/POCD and pro-inflammatory cytokines such as tumor necrosis factor-α, interleukin-1beta, interleukin-6 and interleukin-8, neuronal injury marker and C-reactive protein was shown by several studies and questioned by others (see Sections Biological Markers of Postoperative Delirium and Common Biomarkers of Postoperative Delirium and Cognitive Dysfunction for Details). Some POD/POCD patients have elevation in serum cortisol levels that may be explained by genetic variation of the glucocorticoid receptor gene (Perroud et al., 2011). The isoforms of apolipoprotein E can provoke cholinergic deficiency and acetylcholinesterase unblocking (Soininen et al., 1995), although some results are contradicting. The amyloid beta peptide associated with Alzheimer’s disease was also observed in the serum of POCD patients. These and other findings are discussed in details in Sections Biological Markers of Postoperative Delirium, Common Biomarkers of Postoperative Delirium and Cognitive Dysfunction and Biological Markers of Postoperative Cognitive Dysfunction.

**Figure 2:**
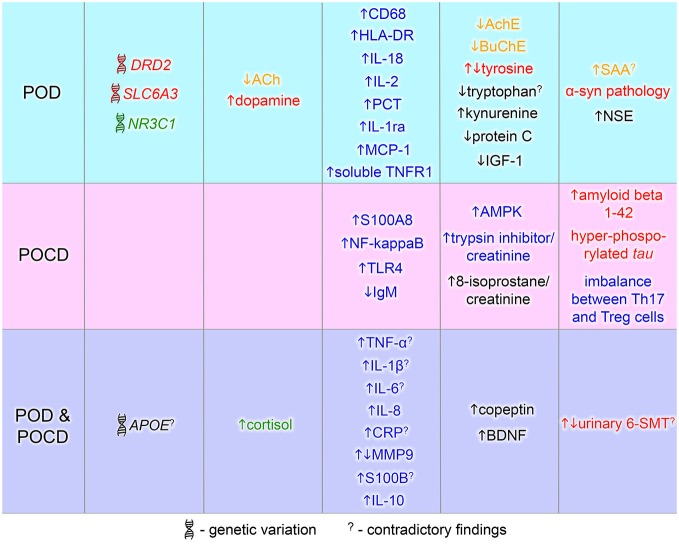
**Biomarkers of postoperative delirium (POD) and postoperative cognitive dysfunction (POCD)**. Biomarkers identified in POD or POCD patients are in blue and pink area respectively. The common POD/POCD biomarkers are presented in the violet area. Font color denotes a marker type: red—dopamine-related marker, green—glucocorticoid-related marker, yellow—cholinergic marker, blue—inflammation-related marker, black—others. 6-SMT, 6-sulfatoxymelatonin; ACh, acetylcholine; AchE, acetylcholinesterase; AMPK, 5′ adenosine monophosphate-activated protein kinase;* APOE*, apolipoprotein E; BDNF, brain-derived neurotrophic factor; BuChE, butyrylcholinesterase; CD68, cluster of differentiation 68; CRP, C-reactive protein; *DRD2*, dopamine receptor D2; HLA-DR, human leukocyte antigen-DR; IGF-1, insulin growth factor-1; IgM, immunoglobulin M; IL, interleukin; MCP-1, monocyte chemotactic protein 1; MMP9, matrix metalloproteinase-9; NF-kappaB, nuclear factor kappa B;* NR3C1*, nuclear receptor family 3, group C, member 1; NSE, neuron specific enolase; PCT, procalcitonin; S100A8, S100 calcium binding protein A8 (myeloid-related protein-8, calgranulin A); S100B, S100 calcium binding protein B; SAA, serum anticholinergic activity;* SLC6A3*, solute carrier family 6, member 3; Th17, T helper 17 cells; TLR4, toll-like receptor 4; TNF-α, tumor necrosis factor-α; TNFR1, tumor necrosis factor receptor-1; Treg, regulatory T cells; α-syn, alpha-synuclein.

Here we review the known genetic risk factors and physiological and immunological changes that have been associated with POD and POCD. Deiner and Silverstein reviewed the postoperative delirium and cognitive dysfunction in 2009. More recent reviews discussed biomarkers and genetic variance for delirium alone (Khan et al., 2011; Stoicea et al., 2014). This article comprises a literature review on both POD and POCD biomarkers with a focus on recent findings. The current knowledge about the contributing biomarkers to postoperative delirium and cognitive dysfunction is summarized in Figure 2. POD and POCD have a wide range of contributing mechanisms and some biomarkers are overlapping. A more detailed description of the known and potentially novel biomarkers is provided below.

## Biological Markers of Postoperative Delirium

The leading hypothesis suggests that delirium can be caused by a central cholinergic deficiency (Inouye, 2006) and is based on treatment with drugs which impair cholinergic function (Tune et al., 1981). The impact on muscarinic anticholinergic burden can be measured by SAA (Plaschke et al., 2007b). Increase of SAA levels is correlated with greater number of delirium symptoms, whereas SAA decrease is correlated with delirium resolution (Mach et al., 1995; Flacker et al., 1998; Mussi et al., 1999). Several studies questioned the association between SAA and delirium, considering that a temporal profile of SAA can be influenced by pre-existing cognitive impairment, infection or illness (Flacker and Lipsitz, 1999; Plaschke et al., 2007a; van Munster et al., 2012). The impact of drugs on the cholinergic system was addressed in detail by Fox et al. (2014) and Praticò et al. (2005).

Acetylcholine plays an important role in memory, associative learning and selective attention (Hasselmo, 1995; Everitt and Robbins, 1997). Impairment of its receptors, such as nicotinic and muscarinic acetylcholine receptors, might lead to cholinergic deficiency and delirium development (Hshieh et al., 2008). Postsynaptic M1 muscarinic receptors are predominantly expressed in hippocampus, cerebral cortex and striatum (Hersch et al., 1994; Levey, 1996) and play a role in cognitive functioning, memory and learning (Anagnostaras et al., 2003; Volpicelli and Levey, 2004; Fisher, 2008). Inhibition of the M1 muscarinic receptor was hypothesized to cause POD and POCD (Praticò et al., 2005). Inhibition of postsynaptic nicotinic receptors by isoflurane and nitrous oxide results in learning and memory impairment after surgery (Culley et al., 2003; Kong et al., 2015). Vice versa, agonists of the nicotinic receptors can improve cognitive function (Wagner et al., 2013): activation of the nicotinic acetylcholine receptor alpha 7 prevents the cognitive decline after surgery by inhibition of NF-kappaB (nuclear factor kappa B) activation and suppression of macrophage migration into the hippocampus (Terrando et al., 2011). This phenomenon shows the bidirectional communication between the nervous and the immune system (Ader et al., 1995). Therefore, acetylcholine and its receptors are likely contributors to the onset of POD and POCD.

The cholinergic anti-inflammatory pathway, mediated by acetylcholine, is associated with neurocognitive decline (Ramlawi et al., 2006). It suppresses NF-kappaB activation and inhibits the release of inflammatory cytokines (e.g., tumor necrosis factor, interleukin (IL)-1β, IL-6, and IL-18) but not IL-10, an anti-inflammatory cytokine (Borovikova et al., 2000; van Gool et al., 2010). Septic and aseptic inflammation can trigger acute cognitive deficits in patients with cholinergic system depletion (Field et al., 2012). Acetylcholinesterase and butyrylcholinesterase inactivate acetylcholine through hydrolysis, possibly enhancing inflammation. Decreased cholinesterase activity in delirious patients was correlated with elevated levels of C-reactive protein and IL-6 (Cerejeira et al., 2012). C-reactive protein (CRP) is a marker of nonspecific acute-phase response in inflammation, infection and tissue damage (Pepys and Hirschfield, 2003), correlated with cognitive decline (Tilvis et al., 2004). The association between high CRP levels and delirium was shown by several studies (Beloosesky et al., 2004; Macdonald et al., 2007; Burkhart et al., 2010; Pol et al., 2014; Ritchie et al., 2014; Zhang et al., 2014b), and questioned by others (Lemstra et al., 2008; Girard et al., 2012).

An inflammatory response to postoperative stress may contribute to delirium via disruption of the blood-brain barrier (Rudolph et al., 2008b). The increased risk is correlated with elevated monocyte chemotactic protein 1, procalcitonin, human leukocyte antigen-DR, CD68, IL-1β, IL-6, IL-8, IL-18 and anti-inflammatory IL-1 receptor antagonist (van Munster et al., 2008, 2011a; van den Boogaard et al., 2011; Cape et al., 2014). Elevated levels of the pro-inflammatory cytokines IL-2 and tumor necrosis factor-α (TNF-α) were detected in the POD patients who had undergone coronary artery bypass graft surgery (Kazmierski et al., 2014a,b). Inhibition of inflammatory IL-12/IL-23-mediated pathways may reduce Alzheimer’s disease pathology and reverse cognitive deficits in aged mice (Vom Berg et al., 2012). van Munster et al. (2011b) observed high levels of IL-8 and cortisol before a delirium onset and high levels of IL-6 and S100 calcium-binding protein B (S100B) in the course of delirium but functional genetic variations in interleukin-6 gene (*IL6*), interleukin-6 receptor gene (*IL6R*) and interleukin-8 gene (*IL8*) were not associated with delirium (van Munster et al., 2011b). Likewise, the association between delirium and higher IL-1, IL-6 and TNF-α plasma levels was not confirmed (Adamis et al., 2007, 2009). The lower plasma concentrations of the coagulation marker protein C together with elevated plasma concentrations of soluble tumor necrosis factor receptor-1 were associated with increased risk for delirium (Girard et al., 2012).

S100B is an indicator of the direct neuronal injury, e.g., by cerebrovascular accidents and traumatic brain injury (Berger et al., 2005). Several studies consistently demonstrated highly elevated levels of S100B in patients with delirium (Pfister et al., 2008; van Munster et al., 2009b, 2010a,c; van den Boogaard et al., 2011). Grandi et al. (2011) found no difference in levels of S100B in delirious and control patients. The same study indicated that neuron-specific enolase and brain-derived neurotrophic factor (BDNF) could be potential biomarkers for delirium in intensive care unit patients (Grandi et al., 2011). BDNF plays role in synaptic plasticity, neuronal survival, differentiation and growth (Acheson et al., 1995; Huang and Reichardt, 2001). Similarly to BDNF, insulin growth factor-1 (IGF-1) promotes neuronal proliferation, development, survival and enhanced synaptic transmission in CNS (Frost et al., 2003; Shcheglovitov et al., 2013; Huat et al., 2014). Tumor necrosis factor-α (TNF-α) can be involved in neurodegeneration through inhibition of IGF-1 (Frost et al., 2003; Bassil et al., 2014). Low baseline levels of IGF-1 were associated with an increased risk of delirium incidence (Wilson et al., 2005; Adamis et al., 2007, 2009). Due to the neuroprotective function, low levels of IGF-1 may have a significant effect on delirium severity (Adamis et al., 2009). Understanding the complex connection between the cholinergic system and increased pro-inflammatory response as well as neurodegeneration is likely to shed light on the molecular and cellular causes of delirium.

Another popular hypothesis suggests that delirium can be caused by dopamine excess and acetylcholine deficiency relative and/or absolute to each other (Trzepacz, 2000). Cytokines can disrupt the neurotransmitter system balance, leading to reduced acetylcholine release (Willard et al., 1999) and increased dopamine and norepinephrine release (Stefano et al., 1994). Delirium, related to anticholinergic mechanisms, was successfully treated with the dopamine receptor antagonists (Alagiakrishnan and Wiens, 2004). One of such receptors is dopamine receptor D2 (DRD2); its dysfunction leads to hallucinations, impairment of motor and frontal lobe functions (Volkow et al., 1998; Makoff et al., 2000). The gene encoding for D_2_ subtype of dopamine receptor (*DRD2*) was associated with schizophrenia and movement disorders (Kukreti et al., 2006; Koning et al., 2012). Seven single nucleotide polymorphisms (SNPs) in the *SLC6A3* (solute carrier family 6, member 3) gene and three genetic polymorphisms in the *DRD2* gene are associated with delirium (van Munster et al., 2010d). The *SLC6A3* gene is coding for the dopamine transporter, hence variation of this gene can lead to a lower concentration of cerebral basal dopamine, diminishing the risk of delirium (van Munster et al., 2010b). One of the detected genetic polymorphisms in *SLC6A3* was associated with pediatric bipolar disorder (Mick et al., 2008); although no connection was found between bipolar disorder in adults and postoperative delirium.

Apolipoprotein E (ApoE) regulates the cholesterol metabolism, participates in repairing and maintaining of neuronal membranes and myelin during development and after injury (Ignatius et al., 1986). It is responsible for cholinergic neuron destruction by increased synthesis and defective clearance of amyloid beta (Kowall et al., 1991). Different isoforms of *APOE* gene can provoke cholinergic deficiency and acetylcholinesterase unblocking (Soininen et al., 1995). The carriers of *APOE* ε4 allele have greater risk of delirium development (Adamis et al., 2007; Leung et al., 2007; van Munster et al., 2009a) and are more predisposed to cellular damage within the brain (Olivecrona and Koskinen, 2012). The *APOE* ε4 allele was found to be correlated with longer duration of delirium in mechanically ventilated critically ill patients (Ely et al., 2007). There might be a connection between neurodegeneration due to pro-inflammatory response and enhanced *APOE* activity that causes cholinergic deficiency in POD patients. Even so, some studies question the association of *APOE* ε4 with delirium (Adamis et al., 2009; Bryson et al., 2011; Abelha et al., 2012).

Elevated serum cortisol levels were correlated with POD risk and degree, being dependent on hypothalamic-pituitary-adrenal axis hyperactivity at preexisting cognitive and functional impairment (van Munster et al., 2010a; Bisschop et al., 2011; Cerejeira et al., 2013; Colkesen et al., 2013; Kazmierski et al., 2013). A possible epigenetic explanation of cortisol sensitivity is the methylation of the glucocorticoid receptor gene *NR3C1* (nuclear receptor family 3, group C, member 1; Perroud et al., 2011). The increased diurnal cortisol and higher sensitivity to glucocorticoids were associated with homozygous *NR3C1* haplotype 4 (Manenschijn et al., 2011). The carriers of this haplotype had a 92% decreased risk of developing POD independently of age, cognitive and functional state (Manenschijn et al., 2011). This study concluded that development of delirium and its pathogenesis is correlated with glucocorticoid signaling. High levels of glucocorticoids affect working memory and thereby explain the cognitive deficits and inattention (Lupien et al., 1999). Mild cognitive impairment was further associated with increased cortisol levels and POD risk (Kazmierski et al., 2014b).

The neurometabolic pathway facilitates communication between brain and metabolic organs and consequently influences various neurodegenerative disorders, normal and pathophysiological aging (Siddiqui et al., 2012). Alteration in the neurometabolic status of the hippocampus can potentially impair growth and survival of neuronal cells, which is a common neuropathology of Alzheimer’s disease (Wenk, 2003; Cong et al., 2013). Metabolic syndrome (e.g., hyperglycemia, diabetes) together with inflammation can contribute to cognitive decline (Yaffe et al., 2004). Prevention of metabolic syndrome by preoperative conventional glucose control might reduce the incidence of POD/POCD (Yaffe et al., 2004; Finfer et al., 2009).

The changes of amino acid concentrations in serum and urine have been associated with POD pathogenesis. An increased risk of delirium development was associated with decreased plasma tryptophan and the ratio of tryptophan as well as the increased or decreased ratio of tyrosine to large neutral amino acids (van der Mast et al., 2000; Robinson et al., 2008; Pandharipande et al., 2009). It was hypothesized that high levels of tyrosine lead to dopamine and norepinephrine excess that are involved in delirium pathogenesis (Pandharipande et al., 2009).

The low tryptophan levels might be associated with delirium via decreased synthesis of brain serotonin (van der Mast et al., 2000; Robinson et al., 2008; Pandharipande et al., 2009). de Jonghe et al. (2012) questioned the association between lower levels of tryptophan and delirium. Following the inflammatory response, tryptophan catabolisation via the kynurenine pathway is increased (Adams Wilson et al., 2012). Elevated plasma kynurenine and kynurenine/tryptophan ratio were correlated with fewer days without acute brain dysfunction in form of delirium or coma (Adams Wilson et al., 2012). Another suggested connection of tryptophan with delirium is alteration of melatonin production via serotonin synthesis (Pandharipande et al., 2009). Melatonin participates in regulation of circadian rhythms and quality and duration of sleep (Brzezinski, 1997). POD patients frequently have disrupted sleep-wake cycle, decreased delta melatonin concentrations (Yoshitaka et al., 2013) and abnormal circadian postoperative patterns of melatonin secretion (Shigeta et al., 2001).

Sunwoo et al. (2013) observed a higher frequency of normal and phosphorylated α-synuclein-positive pathologies in 16 delirious patients that underwent gastrostomy for stomach cancer. Sunwoo and colleagues concluded that POD clinical characteristics are analogous to the core features of α- as dementia with Lewy bodies, Parkinson disease dementia; patients experience altered sleep-wake cycles, visual hallucinations, disorganized thinking and attention impairment (Sunwoo et al., 2013). α-synuclein may be involved in the neurotransmitter release controlling through the SNARE complex (Kang et al., 1987; Tanzi et al., 1987). The delirious state is strongly influenced by the balance between cholinergic and dopaminergic systems, pro-inflammatory signaling, apolipoprotein E isoform, glucocorticoid signaling and the neurometabolic state. Many additional contributors at genetic, proteomic, metabolic and immune levels are to be expected.

## Common Biomarkers of Postoperative Delirium and Cognitive Dysfunction

Postoperative delirium correlates with early postoperative cognitive dysfunction (at 7 days; Rudolph et al., 2008a; Hudetz et al., 2009) and delirious patients have 14 times greater chance of POCD development (Hudetz et al., 2009). In this chapter, we will discuss the common biomarkers found in both cognitive impairments, which are summarized in Figure 2.

Among the above-discussed genetic markers, the *APOE* ε4 allele was associated with greater risk to develop postoperative delirium (Adamis et al., 2007; Leung et al., 2007; van Munster et al., 2009a) and cognitive dysfunction at 7 days postoperatively (Cao et al., 2014). The association with POCD was however not detected by other studies of APOE ε4 variation at 1 week, 1–3 months and 1 year postoperatively (Abildstrom et al., 2004; Rentowl and Hanning, 2004; McDonagh et al., 2010; Bryson et al., 2011; Cao et al., 2014).

The elevated cortisol levels were detected in both POD (van den Boogaard et al., 2011; Cerejeira et al., 2013) and POCD patients (Zhang et al., 2014a). The magnitude of cortisol elevation correlated with levels of anti-inflammatory cytokine IL-10 and pro-inflammatory cytokine IL-6. Similarly to POD, POCD is associated with elevation of other pro-inflammatory markers including IL-1β, IL-8 and TNF-α (Rothenburger et al., 2001; Hudetz et al., 2011; Li et al., 2012; Bi et al., 2014). TNF-α stimulates IL-1β production in the brain and causes postoperative cognitive decline via peripheral cytokine cascade (Terrando et al., 2010). Reducing IL-1 release by peripheral TNF-α blockade might prevent POD, POCD and neuroinflammation (Terrando et al., 2010). Nonspecific acute-phase response in inflammation is present during POD and POCD. POCD patients have elevated levels of CRP following coronary artery bypass grafting (Hudetz et al., 2011), liver transplantation (Li et al., 2013b) and lumbar discectomy (Zhang et al., 2014a). Contradictory to previous findings, plasma levels of inflammatory marker matrix metalloproteinase-9 were decreased in POD patients and elevated in POCD patients (Girard et al., 2012; Zhang et al., 2014a).

Elevated levels of S100B were associated with POD (Pfister et al., 2008; van Munster et al., 2009b, 2010a,c; van den Boogaard et al., 2011). Likewise, POCD patients have increased serum levels of S100B, which is an indicator of neuronal injury (Rasmussen et al., 2000; Li et al., 2012; Lili et al., 2013). S100B-induced neuroinflammation mediates the RAGE (receptor for advanced glycation end product) signaling in microglia (Bianchi et al., 2007). The RAGE signaling pathway may up-regulate pro-inflammatory cytokines via NF-kappaB signaling, indicating its possible role in surgery-induced cognitive decline pathogenesis (Li et al., 2013a). Yet McDonagh et al. (2010) did not find an association between POCD and S100B or CRP.

BDNF showed correlation with POD occurrence in patients (Grandi et al., 2011) and POCD occurrence in aged mice (Tian et al., 2015). It was associated with other neuropsychiatric disorders such as schizophrenia, depression, bipolar disorder and has been suggested as early marker of brain injury (Chiaretti et al., 2003; Muglia et al., 2003; Teixeira et al., 2010).

Copeptin is correlated with severity of the illness and is presumed to be a prognostic measure of outcome prediction in acute illness (Katan and Christ-Crain, 2010). Postoperative plasma copeptin level can be an independent predictor of POD and POCD after coronary artery bypass graft surgery (Dixson et al., 2014). This study observed higher levels of postoperative copeptin in POD and POCD patients compared to controls.

A significant fluctuation of urinary 6-sulfatoxymelatonin (6-SMT), a major metabolite of melatonin, was detected in POCD patients compared to controls (Wu et al., 2014). Clinical subtypes of POD are differently related to the urinary levels of 6-sulfatoxymelatonin: hypoactive patients have higher 6-SMT, whenever hyperactive patients have lower 6-SMT (Balan et al., 2003). However, the association between melatonin and delirium has been challenged by independent studies after failure to confirm these findings (de Jonghe et al., 2014).

As mentioned above, postoperative delirium and cognitive dysfunction may have common contributing factors and biomarkers such as apolipoprotein E isoforms, cortisol signaling, pro-inflammatory cytokines, neurodegenerative marker S100B, copeptin and 6-sulfatoxymelatonin levels.

## Biological Markers of Postoperative Cognitive Dysfunction

Patients with postoperative cognitive dysfunction display biomarkers distinct from delirious patients, which might be related not only to pathology but also postoperative time. The majority of the detected POCD biomarkers are related to inflammation. A recent study reported a positive association between the pro-inflammatory protein S100A8 and POCD development (Lu et al., 2015) and imbalance between T helper 17 cells, a pro-inflammatory subset of CD4^+^T cells, and regulatory T cells, an anti-inflammatory subset of CD4^+^T cells, was observed in POCD patients (Tian et al., 2015).

Postoperative cognitive dysfunction can be predicted by lower preoperative endotoxin immunity following cardiac surgery (Mathew et al., 2003). Lower preoperative levels of immunoglobulin M (anti-endotoxin core antibody) are associated with the greater incidence and severity of POCD (Mathew et al., 2003). A similar study by Rothenburger et al. (2001) suggested an association between lower levels of immunoglobulin M and elevated levels of endotoxin together with IL-8 (Rothenburger et al., 2001).

5′ adenosine monophosphate-activated protein kinase (AMPK) protects CNS by inhibition of inflammatory responses through various mechanisms, including NF-kappaB pathway (Sag et al., 2008; Salminen et al., 2011). This pathways includes NF-kappaB activation by chemokines, cytokines or adhesion molecules and activation of inflammatory cytokines IL-1 and TNF-α (Renard et al., 1997; Chandel et al., 2000). A significant elevation of NF-kappaB, IL-1β and AMPK was shown to result in Toll-like receptor 4 signaling on microglia in the hypothalamus of a POCD rat model (Wang et al., 2013; Bi et al., 2014). Interleukin-1β and NF-kappaB levels gradually decreased over postoperative days (Wang et al., 2013; Bi et al., 2014). Interleukin-1β can be a viable target to interrupt the POCD pathogenesis, as IL-1β-mediated inflammation was triggered by peripheral surgery-induced innate immune response (Cibelli et al., 2010). Another study demonstrated experimentally that inhibition of IL-1 receptors prevents development of POCD and neuroinflammation (Barrientos et al., 2012).

Le et al. (2014) suggested that hippocampus impairment leads to POCD development after they observed a significant reduction of neuronal dendritic spines and neuroinflammation signified by activated microglia, elevation of TNF-α and interleukin-1β in the hippocampi of aged rats. Amyloid beta 1–42 oligomers can impair cognitive and metabolic processes in the hippocampus (Pearson-Leary and McNay, 2012). The elevated levels of amyloid beta 1–42 were associated with a cognitive impairment caused by its interference with insulin signaling in the hippocampus (Pearson-Leary and McNay, 2012). The amyloid hypothesis suggests that amyloid beta peptide is deposited in the brain of Alzheimer’s dementia patients and can form the senile plaques that perturb various signaling mechanisms (Cras et al., 1991). Old mice that developed short-term POCD upon abdominal surgery had Alzheimer’s dementia-like changes: gliosis in brain, enhanced transcriptional and translational activity of the β-amyloid precursor protein, enhanced production of amyloid beta peptide, and hyper-phosphorylation of *tau* in the hippocampus (Wan et al., 2010). POCD patients after liver transplantation had significantly elevated levels of serum amyloid beta peptide, suggesting similar mechanisms as in Alzheimer’s disease (Li et al., 2013b).

Urinary biomarkers could be promising diagnostic and prognostic indicators of postoperative cognitive dysfunction. A high ratio of trypsin inhibitor/creatinine was suggested to be an independent risk factor of POCD in lumbar discectomy patients (Zhang et al., 2014a). Urinary excretion levels of 8-isoprostane/creatinine were elevated as well in POCD patients at 7 days postoperatively compared to control patients (Cheng et al., 2013).

The major POCD biomarkers include inflammation-related molecules, imbalance between pro- and anti-inflammatory signaling and metabolic levels in urine.

## Potential Biomarkers of Postoperative Delirium and Cognitive Dysfunction

At present, there is no standard biomarker for diagnosis and prognosis of postoperative cognitive impairments. Some findings on biomarker association with POD/POCD are contradictory. Thus, generation of genomic, proteomic and metabolomics data as well as implementation of imaging techniques such as MRI are required. In this section, we review the potential biomarkers possibly involved in occurrence and/or progression of postoperative delirium and cognitive dysfunction.

S100A8 (myeloid-related protein-8, calgranulin A), S100A9 (myeloid-related protein-14, calgranulin B), S100A12 (EN-RAGE, calgranulin C) are reliable markers of inflammation (Foell et al., 2004) and potential markers of plaque instability (Abbas et al., 2012). Inhibition of S100A9 significantly improved learning and memory, and reduced neuropathology of Alzheimer’s disease (Chang et al., 2012). Thus, it is promising to investigate potential connection between the calgranulins and POD/POCD.

As mentioned above, inflammation is associated with both POD and POCD. The pro-inflammatory cytokine IL-18 was not studied yet in the context of POD/POCD. Alzheimer’s patients have increased levels of IL-18 in different regions of the brain (Ojala et al., 2009). Being co-localized with *tau*-protein and amyloid beta plaques, IL-18 mediates the hyperphosphorylation of *tau* (Ojala et al., 2009; Sutinen et al., 2012). IL-18 can influence the integrity of neurons and increase neuroinflammation in the brain (Bossù et al., 2010; Sutinen et al., 2012), thus contributing to cognitive decline in Alzheimer’s disease (Bossù et al., 2008). IL-18 receptor complex (IL-18Rα/β) expression is perturbed in preclinical state of mild cognitive impairment and Alzheimer’s disease (Salani et al., 2013). Specifically, IL-18Rα might play role in autoimmune brain damage (e.g., encephalomyelitis) via production of IL-17-producing T helper cells (Gutcher et al., 2006). A splice variant of IL-18Rβ encodes a putative truncated soluble protein that might be a regulator of IL-18 functioning (Andre et al., 2003).

SIGIRR (also called TIR8) is a potential inhibitor of pro-inflammatory IL-18, IL-1 and Toll-like receptor signaling (Thomassen et al., 1999; Wald et al., 2003; Mantovani et al., 2004). The anti-inflammatory effect of SIGIRR might be extended to the brain, as it inhibits inflammation in cooperation with IL-1F5 (a potential anti-inflammatory cytokine; Costelloe et al., 2008). TIGIRR receptor might be an accessory chain for mature IL-37a (Boraschi et al., 2011). IL-37a isoform is exclusively located in the brain and might be a potential anti-inflammatory cytokine (Boraschi et al., 2011). Another isoforms of IL-37 can bind to IL-18Rα and IL-18-binding protein, enhancing IL-18 inhibition (Boraschi et al., 2011).

*IL1RAPL* (IL-1 receptor accessory protein-like) gene was identified as a X-linked mental retardation locus (Carrié et al., 1999). *IL1RAPL* gene encodes a protein homologous to the IL-1/Toll receptor family. Patients with cognitive impairment had a nonsense mutation and deletions in *IL1RAPL* gene (Carrié et al., 1999). *IL1RAPL* gene might have a potential role in memory and learning functioning due to its over-expression in brain structures responsible for memory development such as hippocampus, dentate gyrus and entorhinal cortex (Carrié et al., 1999).

Several studies have reported a possible disruption of the blood-brain barrier integrity during POD (Pfister et al., 2008; Rudolph et al., 2008b). Blood-brain barrier disruption is associated with cognitive, behavioral and mood disturbances (Shalev et al., 2009). Zonulin is a protein that modulates the intercellular tight junction integrity and increases blood-brain barrier permeability (Fasano et al., 2000). Zonulin is involved in movement of macromolecules, fluid and leukocytes between intestinal lumen and bloodstream (Lu et al., 2000; Fasano, 2011). Since zonulin can increase intestinal and bovine brain microvessel endothelial cells permeability, the elevated circulating levels of zonulin can indicate blood-brain barrier pathologies (Karyekar et al., 2003; Fasano, 2011). Zonulin has been already associated with several diseases: celiac disease (Fasano et al., 2000; Fasano, 2011), schizophrenia (Wan et al., 2007), Devic’s disease (Bai et al., 2009), multiple sclerosis (Takeoka et al., 1983) and Guillain-Barré syndrome (Jin et al., 2007; Yang et al., 2008).

Cholinergic-nicotinic genes can be implicated in POD/POCD pathology. Genetic variation within exon 5 of the α4 subunit of nicotinic acetylcholine receptor (*CHRNA4*) gene can modulate the attention network function (Winterer et al., 2007) and was implicated in nicotine dependence (Feng et al., 2004; Li et al., 2005). Several rare *CHRNA4* SNPs were negatively associated with nicotine dependence indicating its protective effect (Wessel et al., 2010; Xie et al., 2011). Nicotine can improve attention, memory and efficiently treat cognitive impairments (Rezvani and Levin, 2001). Patients with genetic variation of *CHRNA4* might abuse nicotine as self-medication of attention deficits in autosomal dominant nocturnal frontal lobe epilepsy (Hirose et al., 1999; Cho et al., 2003), schizophrenia (Winterer et al., 2007; Winterer, 2010) and attention deficit/hyperactivity disorder (Lambert and Hartsough, 1998). In addition, variants on the *CHRNA5-CHRNA3-CHRNA4* gene cluster, implicated in nicotine dependence, are associated with cognitive performance (Winterer et al., 2010).

## System Biology Approaches for Biomarker Discovery

**Figure 3:**
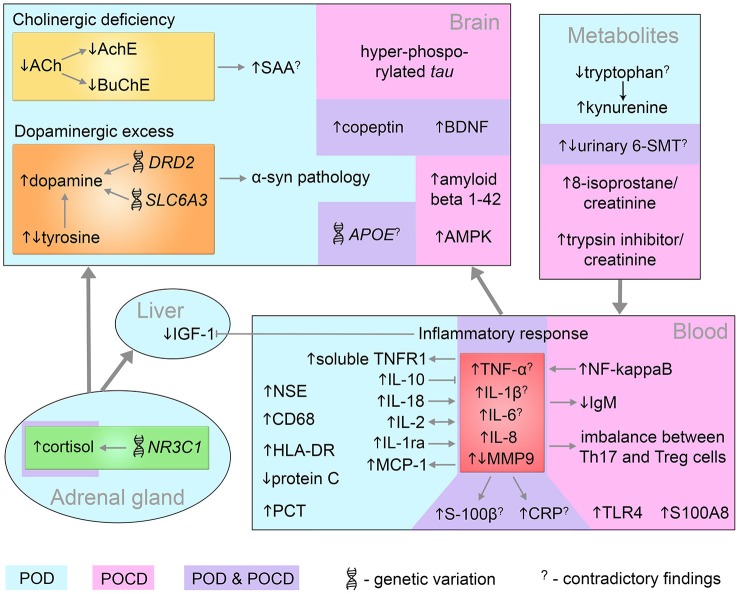
**Systems-level interaction of POD and POCD biomarkers**. Biomarkers identified in POD and POCD patients are in blue and pink area respectively. The common POD/POCD biomarkers are presented in the violet area. 6-SMT, 6-sulfatoxymelatonin; ACh, acetylcholine; AchE, acetylcholinesterase; AMPK, 5′ adenosine monophosphate-activated protein kinase;* APOE*, apolipoprotein E; BDNF, brain-derived neurotrophic factor; BuChE, butyrylcholinesterase; CD68, cluster of differentiation 68; CRP, C-reactive protein; *DRD2*, dopamine receptor D2; HLA-DR, human leukocyte antigen-DR; IGF-1, insulin growth factor-1; IgM, immunoglobulin M; IL, interleukin; MCP-1, monocyte chemotactic protein 1; MMP9, matrix metalloproteinase-9; NF-kappaB, nuclear factor kappa B;* NR3C1*, nuclear receptor family 3, group C, member 1; NSE, neuron specific enolase; PCT, procalcitonin; S100A8, S100 calcium binding protein A8 (myeloid-related protein-8, calgranulin A); S100B, S100 calcium binding protein B; SAA, serum anticholinergic activity;* SLC6A3*, solute carrier family 6, member 3; Th17, T helper 17 cells; TLR4, toll-like receptor 4; TNF-α, tumor necrosis factor-α; TNFR1, tumor necrosis factor receptor-1; Treg, regulatory T cells; α-syn, alpha-synuclein.

To identify, prevent or treat postoperative delirium and cognitive dysfunction we need to connect the incidental findings into an encompassing model and relate the pathomechanisms underlying POD/POCD with clinical outcomes. The biomarkers discussed so far are conceptually linked by the known molecular interactions and pathways and illustrated in Figure 3. Many findings are contradictory between cohorts and studies, which further complicates the investigation of underlying mechanisms. The toolbox of integrated systems biology can help to model the complex dependencies and conceptualize the unknown pathomechanisms contributing to POD/POCD origin and progression. Due to the sparse knowledge on cognitive impairments, we are limited in the choice of methodologies. Predictions of novel targets for study cannot utilize the simulations as appropriately large training and test data needs yet to be collected.

Knowledge maps formed by an integration of large-scale experimental data and text-mining results enable specialists to collaborate on highly detailed information. The dissection of the knowledge map into functionally/pathway enriched modules can reduce the overall complexity and indicate the sub-network(s) deregulated in POD/POCD. Networks built on the list of the seed genes/proteins reviewed in this article may indicate enriched pathways that are related directly or indirectly to POD/POCD. We can proceed with prediction of the upstream regulators, hubs and bottlenecks of the given pathways and sub-networks. Such regulators are potentially interesting as targets since they could modulate the network state and dampen imbalance and deregulation. Network approach enables us to study dynamical changes of the system such as responsiveness, adaptation and stability. For example, network analysis of the metabolic positron emission tomography scans from Parkinson’s disease patients identified two distinct disease-related patterns (Eckert et al., 2007). One of the patterns is related to motor manifestations of Parkinson’s disease, the other pattern is correlated with the patients’ performance on memory and executive functioning tests. In case of POD/POCD, networks can initially be based on the literature mining results where large-scale human experimental data is not accessible. Networks based on experimental data and supported by literature evidence may give stronger results and reflect the network dynamics.

Systems biology methods, applied to Parkinson’s disease, made a significant contribution for integration of known pathomechanisms. Parkinson’s disease is a multi-factorial condition with complex interplay between genetic and environmental factors (Calne et al., 1986). A recently published Parkinson’s disease map is able to capture the known contributing mechanisms, integrate the underlying pathways and visualize large experimental data on top of the solid, literature derived and reviewed network (Fujita et al., 2014). The principle of the Parkinson’s disease map could be well applied to investigate other complex diseases including POD and POCD.

Magnetic resonance imaging revealed the vulnerable regions in brain of POD patients (Root et al., 2013) as well as white-matter hyperintensities (Hatano et al., 2013) and brain atrophy (Gunther et al., 2012). Integration of imaging results with information at different levels (i.e., DNA, RNA, proteins, etc.) gives a rise to mathematical/computational modeling of POD and POCD states. Iterative prediction and cross-validation steps improve such models and system behavior and response to perturbations can be predicted. For instance, neuroimaging integration with genetic and demographic information by a Support Vector Machine algorithm successfully differentiated Alzheimer disease and mild cognitive impairment from controls (Kohannim et al., 2010). The integrated systems biology approaches in the context of POD/POCD lead a step forward to personalized medicine and effective clinical trials.

## Conclusion

Postoperative delirium and cognitive dysfunction has been elucidated on the molecular basis and many biomarkers have been identified. Hypotheses to explain the major features of the disease onset and pathology were formulated but at this point, we understand little how much the markers and mechanisms explain the pathology. In particular, we know little about possible molecular influences on POD/POCD sub-types such as slow and fast progression or hypo- and hyperactive delirium. Common molecular mechanisms with other syndromes, in particular schizophrenia, promise further insights but have not been investigated on a sufficient scale. As neuropsychiatric syndromes present themselves in the most complex manner, we require global standardization efforts and patient cohorts for comparative investigations.

We have reviewed the knowledge about molecular mechanisms underlying POD and POCD and described many biomarkers associated with these postoperative complications, and it is clear that postoperative delirium and cognitive dysfunction are multifactorial conditions. Among the identified pathomechanisms, some biomarkers were common, such as elevation of TNF-α, interleukin-1β, interleukin-6, interleukin-8, interleukin-10, CRP, S100B, matrix metalloproteinase-9, BDNF, copeptin and cortisol levels as well as presence of ApoE ε4 allele. The application of integrated systems biology approaches may elucidate the unknown pathomechanisms contributing to POD/POCD origin and progression. Combining experimental measurements, imaging techniques and mathematical/computational modeling can give a potential to reconstruct the underlying network of molecular interactions and predict reliable biomarkers of postoperative delirium and cognitive dysfunction.

### PubMed Search Strategy

We reviewed the pertinent literature retrieved by a search in the PubMed database (on November 20, 2014) using the following query: “(biomarker OR marker) AND [(postoperative delirium) OR delirium OR (postoperative cognitive dysfunction) OR POCD] AND (hasabstract[text] AND Humans[Mesh]) NOT (Alzheimer OR Parkinson)”. The search yielded 254 publications. The ones cited in the review are those that, in the author’s view, make a substantial contribution to the knowledge about existing and potential biomarkers of POD/POCD.

## Conflict of Interest Statement

The authors declare that the research was conducted in the absence of any commercial or financial relationships that could be construed as a potential conflict of interest.
